# IL-8/CD181 Mediated Inflammation in SLE-Associated Hemolytic Anemia 

**DOI:** 10.30699/ijp.2025.2051460.3403

**Published:** 2025-03-10

**Authors:** Muslim Idan Mohsin, Samer A. MH. Al-Hilali, Rusul Idan Mohsin, Mohammed Mohasin, Mohammed Jasim Mohammed Al-Shamarti

**Affiliations:** 1 *University of Kufa, Faculty of Science, * *Pathological* * Laboratory Analysis Department, Najaf, Iraq*; 2 *Jabir Ibn Hayyan University for Medical and Pharmaceutical Sciences, Najaf, Iraq *; 3 *University Of Dhaka, Department of Biochemistry and Molecular Biology, Dhaka, Bangladesh*

**Keywords:** SLE, IL-8, CD181, CXCR1, hemolytic anemia and mRNA

## Abstract

**Background & Objective:**

Systemic Lupus Erythematosus (SLE) is an autoimmune disease characterized by immune dysregulation, autoantibody production, and organ damage, notably in the kidneys. Cytokine imbalances contribute to SLE's diverse clinical presentations. This study investigated the roles of interleukin-8 (IL-8) and its receptor, CD181 (CXCR1), in SLE pathogenesis, specifically focusing on their association with hemolytic anemia, a severe complication.

**Methods:**

This research investigates the role of interleukin-8 (IL-8) and its cognate receptor, CXCR1 (CD181). It was analyzed clinical and demographic data from 250 SLE patients and quantified IL-8 and CD181 mRNA and protein expression in samples from patients with active SLE, inactive SLE, and SLE complicated by hemolytic anemia, comparing them to healthy controls.

**Results:**

Of the 250 samples, 84% were from SLE patients, with 67% in the active disease phase. Significant upregulation of both IL-8 and CD181 mRNA and protein was observed in SLE patients compared to controls. Specifically, mRNA expression was significantly elevated in active SLE (p=0.0001) and inactive SLE (p=0.01). Notably, IL-8 mRNA expression was significantly higher in SLE patients with hemolytic anemia (p<0.0001) compared to those without (p<0.01(.

**Conclusion:**

These findings suggest that the IL-8/CD181 axis plays a crucial role in the inflammatory processes and tissue damage associated with SLE, particularly in the development of hemolytic anemia.

## Introduction

Systemic Lupus Erythematosus (SLE) is a heterogeneous autoimmune disorder of unknown etiology that affects multiple organ systems. In SLE, autoantibodies produced by the immune system mistakenly attack healthy cells and tissues, resulting in inflammation and multisystem organ damage (1). The exact cause of SLE remains unclear; however, a complex interplay of genetic predisposition, environmental factors (such as ultraviolet light and infections), and hormonal influences is believed to disrupt immunological tolerance to self-antigens, leading to tissue and organ injury (2).

The annual incidence of SLE is approximately 4 cases per 100,000 persons, with a prevalence of 73 cases per 100,000 (3). The clinical presentation of SLE is highly variable, both among different individuals and within the same individual over time, leading to a protean disease course that ranges in severity from mild and indolent to rapidly progressive and life-threatening (4). Some patients may present with arthritis and cutaneous manifestations, while others experience debilitating fever, fatigue, arthralgia, or severe organ involvement (5).

Diagnosis is based on a combination of positive serologic findings and characteristic clinical features. However, diagnosis can often be delayed or challenging, requiring careful clinical judgment to correlate serologic data with clinical presentation (6). Diagnostic criteria include immunologic biomarkers and involvement of various organ systems—such as mucocutaneous, musculoskeletal, hematologic, renal, and central nervous system features—along with positive antinuclear antibody (ANA) tests (7, 8).

Standard treatment for SLE includes hydroxychloroquine (HCQ), glucocorticosteroids, and immunosuppressive agents. Belimumab, a biologic agent, is the first approved biologic therapy for SLE (9). Persistent disease activity and medication-related toxicity significantly contribute to the risk of irreversible organ damage and increased mortality (10, 11).

SLE is characterized by a breakdown in self-tolerance, regulated by mechanisms involving T-cell competition, effector cell dysfunction, and abnormal cell death (12). T cells play a pivotal role in the immunopathogenesis of SLE by supporting B-cell activation, promoting their differentiation and proliferation, facilitating immunoglobulin class switching, and ultimately enhancing the production of autoantibodies (13).

Interleukin-8 (IL-8), a chemokine primarily secreted by macrophages and neutrophils, plays a crucial role in the inflammatory response and tissue injury (14). Elevated IL-8 levels have been observed in the cerebrospinal fluid of patients with neuropsychiatric SLE. Additionally, increased levels of CXCR1, a receptor for IL-8, have been detected in patients with active, but not remissive, SLE (15). The heightened production of IL-8 contributes to the recruitment of neutrophils and other immune cells, which may worsen hemolysis observed in autoimmune hemolytic anemia (AIHA) through the release of further inflammatory mediators (16).

Objective: This study aimed to assess mRNA and plasma levels of IL-8 and its receptor (IL-8R) in patients with SLE and to examine their correlation with disease activity and association with hemolytic anemia. We hypothesized that IL-8 and IL-8R expression levels vary according to disease status—active versus inactive disease, and presence versus absence of hemolytic anemia. This study quantitatively evaluated the relationship between CD181 (IL-8R) and IL-8 levels in relation to SLE activity, with a particular focus on patients exhibiting hemolytic anemia.

## Materials and Methods

### Sample Collection

Samples were collected between November 1, 2023, and September 22, 2024. A total of 250 blood samples were analyzed, comprising 180 from SLE patients and 70 from healthy controls, stratified by disease activity (active vs inactive) and the presence or absence of hemolytic anemia. Both mRNA and protein levels of CD181 and IL-8 were assessed using real-time quantitative reverse transcription PCR (qRT-PCR) and enzyme-linked immunosorbent assay (ELISA), respectively.

All SLE cases were diagnosed in accordance with the American College of Rheumatology (ACR) classification criteria (17). The subjects were grouped into three categories: active disease, inactive (remission), and healthy controls. For each patient, two samples were collected. Whole blood samples were immediately frozen post-collection. Trizol was added prior to freezing to preserve RNA integrity.

### Disease Activity Assessment

The disease activity of SLE patients was evaluated using the Systemic Lupus Erythematosus Disease Activity Index (SLE-DAI), following internationally recognized guidelines. Patients were categorized based on their SLE-DAI scores as follows:

- Remission (no active disease): 0
- Low activity: 1–5
- Moderate activity: 6–10
- High activity: 11–19
- Very high activity: ≥20 (18)

### Laboratory Parameters

A comprehensive serological evaluation was performed on all samples to confirm SLE diagnosis and further stratify patients by disease activity and anemia type (hemolytic or non-hemolytic). The panel included:

- Antinuclear antibody (ANA) and anti–double-stranded DNA (anti-dsDNA) assays (autoimmunity markers)
- Complement components C3 and C4 (indicators of immune complex activation)
- Direct Coombs test (to detect antibody-mediated hemolysis)
- Total serum bilirubin levels (marker of hemolysis)

**Fig. 1:**
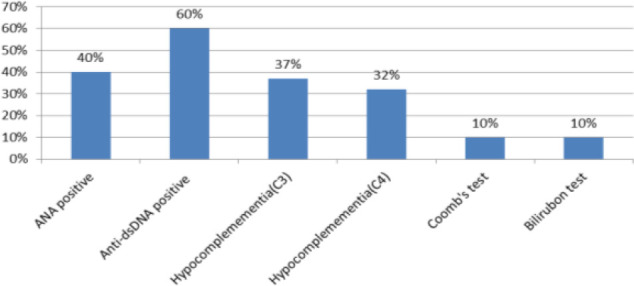
The levels of ANA, anti-dsDNA, complement C4 and C3, coomb's test, and total bilirubin tests for all samples

These findings are summarized in Fig. 1. 

### RNA Extraction and Quantitative PCR Analysis

Whole blood specimens were used to establish the healthy control group and to define the experimental cohorts, consisting of patients with either active disease or disease in remission. Total RNA was extracted from all samples following the manufacturer’s instructions (Solarbio Life Science). After centrifugation at 200 × g for 5 minutes, the cells were lysed, and RNA was purified through sequential washes with RPE buffer, followed by two washes with WT buffer. The purified RNA was then eluted and stored at –20°C until further analysis.

Reverse transcription was performed to synthesize complementary DNA (cDNA). Quantitative PCR (qPCR) was conducted using Primer Design Precision’s 2× SYBR Green Master Mix. Pre-designed primer/probe sets for the genes of interest were used as quality control measures. Amplification was carried out using the Applied Biosystems 7900HT Fast Real-Time PCR System for 40 cycles. Fluorescence emission from the amplified product was used to determine the cycle threshold (Ct), which is inversely proportional to the expression level of the target genes IL-8 and CD181.

The following primer sequences were used:

- CD181 (CXCR1):

- Forward: 5′-CTGATCTCTGACTGCAGCTCCT-3′
- Reverse: 5′-CAGCAATGGTTTGATCTAACTGAAG-3′

- IL-8:

- Forward: 5′-AGTTTTTGAAGAGGGCTGAGA-3′
- Reverse: 5′-TGCTTGAAGTTTCACTGGCATC-3′

- GAPDH (reference gene):

- Forward: 5′-TGCACCACCAACTGCTTAGC-3′
- Reverse: 5′-GGCATGGACTGTGGTCATGAG-3′

All primers were purchased from Macrogen, South Korea. Relative gene expression levels were normalized against the reference gene GAPDH, and the comparative Ct (ΔΔCt) method was used to calculate fold changes in gene expression (19).

### Estimation of Human IL-8 and CD181 Levels

Plasma samples were analyzed to determine IL-8 and CD181 concentrations using a standardized immunoassay. Prior to the assay, samples underwent brief centrifugation to remove particulate matter. A 2000 pg/mL stock standard solution was prepared by reconstituting the standard in 1.0 mL of standard sample diluent. Serial dilutions were subsequently performed by transferring 500 µL of the standard diluent into a tube labeled for 1000 pg/mL, and continuing this serial dilution process across the remaining tubes to construct a standard curve.

The assay procedure involved adding 100 µL of either standard or sample to individual wells of a microtiter plate. Plates were incubated for 90 minutes at 37°C under sealed conditions. Each well was then washed three times with 1× wash buffer through aspiration and replacement. After washing, 100 µL of a biotin-conjugated anti-human IL-8 and CD181 antibody working solution was added to each well, followed by a 30-minute incubation at 37°C. The plate was again washed three times.

Next, 100 µL of horseradish peroxidase (HRP)-avidin working solution was added to each well, and the plate was incubated for an additional 30 minutes at 37°C after another washing cycle. The colorimetric reaction was initiated by adding 100 µL of tetramethylbenzidine (TMB) substrate to each well, followed by gentle mixing and incubation in the dark at 37°C for 15–20 minutes. The reaction was terminated by adding 50 µL of stop solution, and optical density (OD) was measured within 30 minutes using a microplate reader at 450 nm.

The concentrations of IL-8 and CD181 in the unknown samples were calculated by interpolation against the standard curve generated during the assay.

### Statistical Analyses

All statistical analyses and graphical data representations were conducted using GraphPad Prism version 10. One-way ANOVA was employed for intergroup comparisons, with appropriate post hoc tests to adjust for multiple comparisons. Data are presented as mean ± standard error of the mean (SEM). Statistical significance was defined by a predetermined threshold (e.g., *P* < 0.05).

## Results and Discussion

This study aimed to develop a diagnostic approach for systemic lupus erythematosus (SLE) across multiple disease states. A total of 250 samples were prospectively collected from hospitals across Iraq between November 1, 2023, and September 22, 2024, based on predefined inclusion criteria. These included patients with active SLE, patients in remission (inactive disease), and healthy controls. All samples were evaluated for antinuclear antibodies (ANA) and anti–double-stranded DNA (anti-dsDNA) antibodies.

The core diagnostic methodology utilized an enzyme-linked immunosorbent assay (ELISA) on the Chorus/Trio instrument. The assay involved immobilizing the target antigen on a solid-phase support. After incubation with diluted human plasma, specific immunoglobulins bound to the immobilized antigen. Unbound proteins were removed through repetitive washing, followed by detection using an HRP-conjugated anti-human immunoglobulin antibody. A peroxidase substrate was added to initiate a colorimetric reaction, and the color intensity was measured to determine the antibody titer. The Chorus/Trio platform employed disposable, self-contained reagent cartridges for automated processing.

**Fig. 2:**
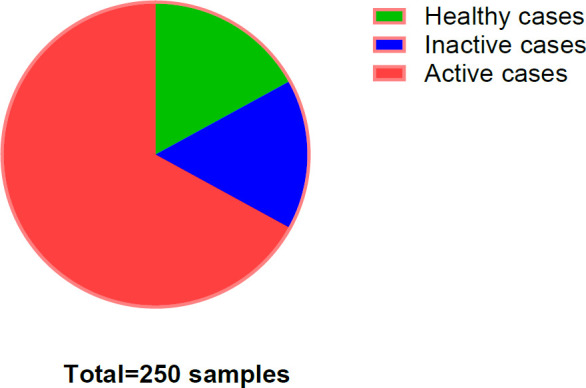
The percentage of different types of SLE disease activity samples that taken from different people

Results were expressed in international units per milliliter (IU/mL) and analyzed for their diagnostic relevance in SLE. As illustrated in Figure 2, approximately 16% of the study cohort comprised healthy controls, while the remaining 84% were SLE patients. Of these, 67% exhibited active disease, with the remainder in remission. The higher proportion of active cases was consistent with previously published findings (20).

 The results present the distribution of 250 samples collected from various SLE cases across multiple hospitals in Iraq. All samples were screened for systemic lupus erythematosus using antinuclear antibody (ANA) and anti–double-stranded DNA (anti-dsDNA) tests.

### The changes in IL-8 and CD181 at the mRNA level in response to SLE disease

 This study aimed to investigate the association between IL-8 expression and different stages of systemic lupus erythematosus (SLE) activity. Total RNA was extracted and reverse transcribed into complementary DNA (cDNA) using a commercially available kit. Relative gene expression was quantified by quantitative PCR (qPCR), and fold change was calculated using the 2^(-ΔΔCt) method.

**Fig. 3:**
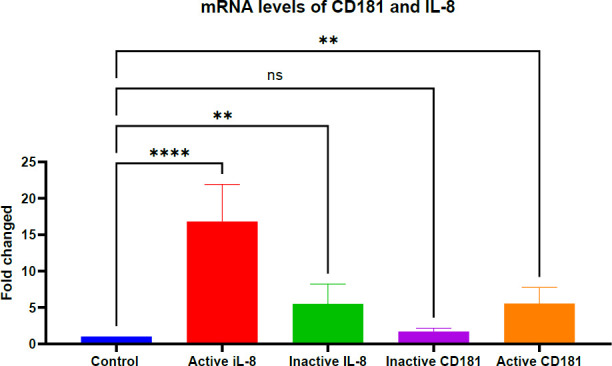
mRNA expression levels of IL-8 and CD181 in different SLE disease states.

As shown in Figure 3, both CD181 and IL-8 expression levels were elevated in patients with active and inactive disease. A statistically significant increase in IL-8 and CD181 expression was observed in the active disease group (*P* = .0001), and a moderate but significant increase was also noted in the inactive disease group (*P* = .01), indicating upregulation in both disease states.

These findings are consistent with prior studies (21), which similarly reported elevated levels of IL-8 and CD181 in SLE patients. The observed upregulation in both active and inactive groups supports the potential of IL-8 and CD181 as biomarkers for SLE, aligning with previous reports (7, 22).

Quantitative PCR (qPCR) was used to assess IL-8 and CD181 expression in whole blood samples from patients with active SLE, patients in remission (inactive disease), and healthy controls. Gene expression was normalized to the housekeeping gene GAPDH, and relative expression levels were calculated using the 2^(-ΔΔCt) method. Results indicate significant upregulation of IL-8 and CD181 in both active and inactive SLE groups compared to healthy individuals. One-way ANOVA was performed to determine statistical significance: *p* < 0.01 (**), *p*<0.0001 (***), and ns indicates non-significant differences. Data represents the mean values from three independent experiments, each conducted using a total of 250 samples.

### Protein Expression of CD181 and IL-8 in SLE Patients

This section aimed to investigate the correlation between protein levels of CD181 and IL-8 and their corresponding gene expression changes across varying clinical presentations of systemic lupus erythematosus (SLE). Biological samples were collected and categorized according to established diagnostic and disease activity criteria. Plasma samples were obtained from healthy controls and SLE patients, and stored at –20°C until analysis.

IL-8 and CD181 protein concentrations in plasma were measured using a commercially available sandwich ELISA kit (Solorbio, China), following the manufacturer’s instructions. Quantification was performed by comparing optical density readings to a standard curve generated using known concentrations of the target proteins.

**Fig. 4:**
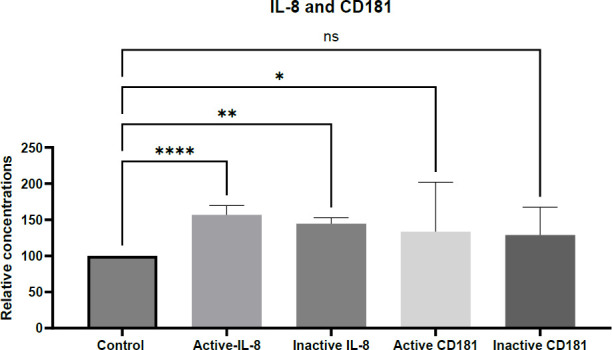
The IL-8 and CD181 proteins expression has changed in response to SLE disease in different cases.

As shown in Figure 4, there was a statistically significant elevation in both CD181 and IL-8 protein levels in active and inactive SLE groups compared to healthy controls (*P* < .05; actual *P* values should be reported where available). These findings are consistent with previously published data (21).

Toll-like receptors (TLRs), particularly TLR7 and TLR8, are often overexpressed in SLE and play a critical role in nucleic acid recognition. Their activation stimulates the production of pro-inflammatory cytokines, including IL-8 and CD181, by various immune cells (5). Both innate and adaptive immune pathways contribute to SLE pathogenesis. IL-8 (CXCL8) production can be triggered by diverse stimuli such as growth factors, bacterial components, and viral infections.

Elevated levels of IL-8 and CD181 were observed across all disease stages—including clinical remission, mild/moderate, and severe disease—suggesting a consistent role for these mediators throughout the SLE disease course. A key observation of this study was the positive association between IL-8 levels and disease activity, evident in both active and inactive lupus, implying a coordinated regulation of IL-8 secretion in SLE.

This coordinated expression may play a critical role in SLE immunopathogenesis, particularly given IL-8’s chemotactic role in recruiting immune cells to sites of inflammation. Although chemokine levels were increased in all SLE groups compared to healthy controls, the difference in IL-8 expression between mild/moderate and severe cases warrants further investigation.

The presence of elevated IL-8 in SLE patients receiving long-term treatment has been previously documented (5), suggesting that standard therapeutic regimens may not sufficiently suppress pro-inflammatory innate immune mediators. These findings support the potential benefit of incorporating targeted chemical inhibitors against specific pro-inflammatory molecules—such as IL-8 and CD181—into existing treatment protocols to improve clinical outcomes in SLE.

The expression levels of IL-8 and CD181 proteins were measured by ELISA in patients diagnosed with active and inactive SLE, and compared with healthy controls. Quantification of IL-8 expression in both healthy and affected individuals was performed using a standard curve method, according to the manufacturer's instructions. Both IL-8 and CD181 showed measurable alterations in plasma.

The significance of observed differences was assessed using one-way ANOVA, with the following statistical thresholds: *p* < 0.05 (), p < 0.01 (**), p <0 .0001 (***), highly significant), and ns for non-significant results. Data represent the means of 250 samples, obtained from three independent experiments, each performed in duplicate.

### IL-8 and CD181 mRNA Expression in SLE-Associated Hemolytic Anemia

This section investigated the modulation of IL-8 and CD181 expression in cases of hemolytic anemia secondary to SLE. Total RNA was extracted and reverse-transcribed into cDNA using a commercial reverse transcription kit. Following qPCR analysis, gene expression levels were quantified using the 2^(-ΔΔCt) method.

The results revealed a notable increase in IL-8 and CD181 mRNA expression in hemolytic anemia cases compared to controls. While both hemolytic and non-hemolytic anemia groups exhibited significantly elevated mRNA levels relative to healthy controls (Figure 4), IL-8 expression was markedly higher in the hemolytic anemia group (*p* < .0001) than in the non-hemolytic group (*p* < .01). CD181 mRNA levels were also significantly elevated in both patient subgroups.

These findings align with earlier reports indicating increased IL-8 levels in both hemolytic and non-hemolytic anemia (16, 23). Specifically, IL-8 and CD181 levels were significantly higher in hemolytic anemia patients than in healthy controls. The more pronounced upregulation of IL-8 suggests a potential role in exacerbating hemolytic anemia in SLE patients.

The functional role of IL-8 in macrophage and neutrophil activity and its contribution to tissue injury has been highlighted by Ghafouri-Fard et al., who reported elevated IL-8 levels in the cerebrospinal fluid of patients with neuropsychiatric SLE. Additionally, increased CCL8 levels have been observed in patients with active, but not remissive, SLE (15). These cumulative findings support the utility of IL-8 and CD181 as potential biomarkers and therapeutic targets for SLE and its associated complications.

However, contrasting findings from Rodriguez-Rosales et al. demonstrated reduced CD181 expression in neutrophils with functional abnormalities, including elevated resting oxidative burst and impaired phagocytic function (24). Such discrepancies may stem from differences in sample type and disease context.

**Fig. 5:**
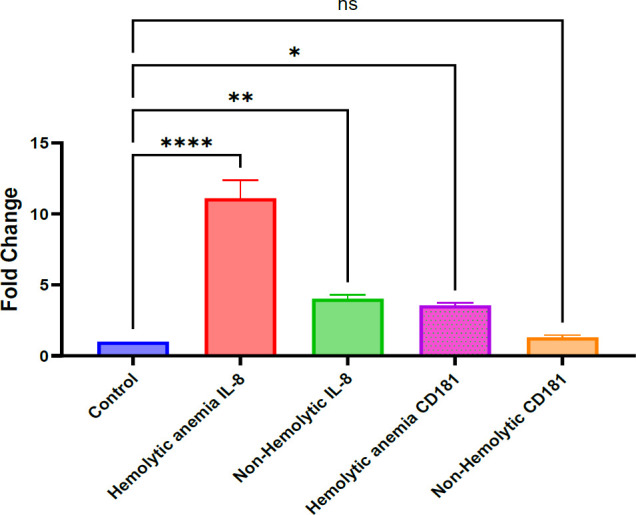
The IL-8 and CD181 mRNA expression has changed in response to SLE with hemolytic anemia and non-hemolytic anemia

IL-8 and CD181 mRNA Expression in SLE Patients With Hemolytic Anemia

IL-8 and CD181 expression levels were measured by quantitative PCR (qPCR) in patients diagnosed with SLE-associated hemolytic anemia and compared with healthy controls. Gene expression was calculated using the 2^–ΔΔCt method, with GAPDH serving as the housekeeping reference gene. Both IL-8 and CD181 showed altered expression in whole blood cells of SLE patients. Statistical significance of the differences was evaluated using one-way ANOVA, with the following thresholds: *p* < .05 (), p < .01 (**), p < .0001 (***, highly significant), and ns for non-significant results. Data represent the means from 250 samples, each assessed in three independent experiments with duplicates.

**Fig. 6:**
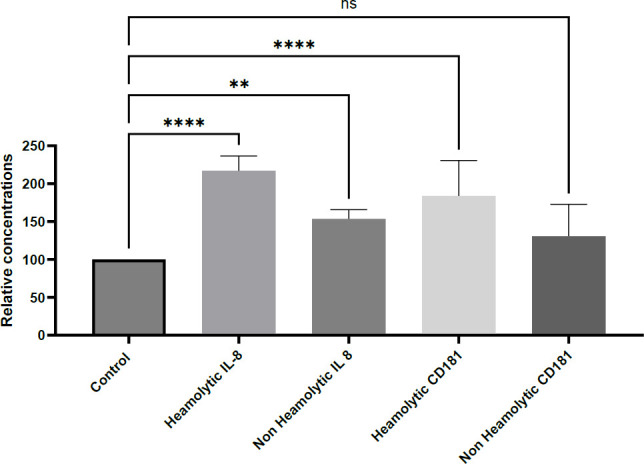
IL-8 and CD181 protein expression levels were altered in response to systemic lupus erythematosus (SLE), with distinct changes observed in patients with hemolytic and non-hemolytic anemia.

### IL-8 and CD181 Protein Levels in SLE-Associated Hemolytic Anemia

This section examined the hypothesis that altered IL-8 and CD181 expression, at both protein and gene levels, is associated with hemolytic and non-hemolytic anemia in systemic lupus erythematosus (SLE). Plasma samples were collected from healthy controls and SLE patients, categorized based on predefined clinical criteria, and stored at –20°C. Plasma concentrations of IL-8 and CD181 were quantified using commercial ELISA kits (Solorbio, China) following the manufacturer’s protocols and the sandwich ELISA method. Quantification was achieved through standard curve analysis.

The results revealed a statistically significant elevation in both IL-8 and CD181 protein levels in SLE patients with hemolytic and non-hemolytic anemia, compared to healthy controls. Notably, a more pronounced increase (*p* < .0001) was observed in the hemolytic anemia group, while IL-8 levels were also significantly elevated in the non-hemolytic group (*p* < .01). In contrast, CD181 levels were significantly increased only in the hemolytic anemia subgroup (Figure 6).

These findings are in agreement with previous studies reporting elevated IL-8 levels in both hemolytic and non-hemolytic anemia (15, 16, 23). It is proposed that the elevated IL-8 expression results from uncontrolled activation of the mononuclear phagocyte system, a hallmark of immune tolerance breakdown, leading to increased IL-8 production. This pro-inflammatory cytokine plays a key role in recruiting neutrophils and other immune cells to sites of inflammation, which may further exacerbate hemolysis in autoimmune hemolytic anemia (AIHA). These recruited cells can intensify erythrocyte destruction by releasing additional inflammatory mediators (23).

Collectively, these data support the hypothesis that IL-8 and CD181 are key contributors to SLE disease activity, especially in cases complicated by hemolytic anemia, and may serve as valuable diagnostic biomarkers or therapeutic targets in this context.

The protein expression levels of IL-8 and CD181 were measured by ELISA in patients diagnosed with SLE-associated hemolytic anemia, and compared with healthy controls. Quantification was performed using the standard curve method, in accordance with the manufacturer’s instructions. Both IL-8 and CD181 showed measurable alterations in plasma.

Statistical significance of group differences was assessed using one-way ANOVA, with the following thresholds: **** *p* < .0001 (highly significant), *p* < .05 (significant), and ns for non-significant results. Data represent the mean values from 250 samples, obtained from three independent experiments, each performed in duplicate.

## Conclusion

This study demonstrates that IL-8 and CD181, at both the mRNA and protein levels, exhibit significant changes across different SLE disease states, particularly in patients with hemolytic anemia. The marked elevation of IL-8 in hemolytic anemia cases (*p* < .0001) underscores its potential utility as a diagnostic and prognostic biomarker.

These findings suggest that incorporating IL-8 and CD181 assessments into routine clinical evaluations could improve early detection and management of SLE, especially in patients at risk of developing hemolytic complications. Moreover, both biomarkers may represent novel therapeutic targets, potentially improving patient outcomes and reducing the overall disease burden.

Further studies are warranted to better understand the underlying mechanisms and to validate these biomarkers in larger, ethnically diverse patient populations.

## Ethical Approval

This research does not contain any studies with human subjects or animals done by any of the authors. 

## Funding/Support

The authors have no discord of interest to declare, and the Funincial support is University of Kufa.

## Data Reproducibility

Data are available upon reasonable request from the corresponding author. 

## Conflict of Interest

The authors declare no conflict of interest. 

## References

[1] Pieterse E, van der Vlag J (2014). Breaking immunological tolerance in systemic lupus erythematosus. Front Immunol..

[2] Katsuyama T, Tsokos GC, Moulton VR (2018). Aberrant T cell signaling and subsets in systemic lupus erythematosus. Front. Immunol..

[3] Filotico R, Mastrandrea V (2018). Cutaneous lupus erythematosus: clinico-pathologic correlation. Giornale italiano di dermatologia e venereologia: organo ufficiale, Societa italiana di dermatologia e sifilografia.

[4] Chen X, Liu H-L (2022). Severe chemosis in lupus. BMJ.

[5] Han X, Li X, Yue SC, Anandaiah A, Hashem F, Reinach PS (2012). Epigenetic regulation of tumor necrosis factor α (TNFα) release in human macrophages by HIV-1 single-stranded RNA (ssRNA) is dependent on TLR8 signaling. J Biol Chem.

[6] Fava A, Petri M (2019). Systemic lupus erythematosus: diagnosis and clinical management. J Autoimmun..

[7] Sota J, Rigante D, Ruscitti P, Insalaco A, Sfriso P, de Vita S (2019). Anakinra Drug Retention Rate and Predictive Factors of Long-Term Response in Systemic Juvenile Idiopathic Arthritis and Adult Onset Still Disease. Front Pharmacol..

[8] AL-Msaid HL, Aledhari M, Alrehbawy R (2024). Investigation of Some Clinical Parameters in Renal Failure Patients. J Biosci Appl Res.

[9] Basta F, Fasola F, Triantafyllias K, Schwarting A (2020). Systemic lupus erythematosus (SLE) therapy: the old and the new. Rheumatol Ther.

[10] Frodlund M, Reid S, Wettero J, Dahlstrom O, Sjowall C, Leonard D (2019). The majority of Swedish systemic lupus erythematosus patients are still affected by irreversible organ impairment: factors related to damage accrual in two regional cohorts. LUPUS.

[11] AL-Msaid HL, Khalfa HM (2019). Relationship between interleukin 17 & 6 in patients with varicocele compare with a control group. Journal of Medical and Life Science.

[12] Woods M, Zou YR, Davidson A (2015). Defects in Germinal Center Selection in SLE. Front Immunol..

[13] Mak A, Kow NY (2014). The pathology of T cells in systemic lupus erythematosus. J Immunol Res.

[14] Mageed AH, Mohsin MI, Al-Sahaf S (2023). One-Pot Multicomponent Synthesis, Antibacterial and Antiproliferative Evaluation of Indole Derivatives. Pharm Chem J.

[15] Ghafouri-Fard S, Shahir M, Taheri M, Salimi A (2021). A review on the role of chemokines in the pathogenesis of systemic lupus erythematosus. Cytokine..

[16] Cheng Y, Sun L, Zou S, Li F, Zhan Y, Wang W ( 2015 ). CXCL13, CCL4, and sTNFR as circulating inflammatory cytokine markers in primary and SLE-related autoimmune hemolytic anemia.

[17] Hochberg MC (1997). Updating the American College of Rheumatology revised criteria for the classification of systemic lupus erythematosus. Arthritis and rheumatism.

[18] Jesus D, Matos A, Henriques C, Zen M, Larosa M, Iaccarino L (2019). Derivation and validation of the SLE Disease Activity Score (SLE-DAS): a new SLE continuous measure with high sensitivity for changes in disease activity. Ann Rheum Dis.

[19] Mohsin MI, Al-Shamarti MJ, Mohsin RI, Al-Sahaf S (2020). Role of Interleukin-36 in Response to Pseudomonas Aeruginosa Infection. Indian Journal of Forensic Medicine & Toxicology.

[20] Elewa EA, Zakaria O, Mohamed EI, Boghdadi G (2014). The role of interleukins 4, 17 and interferon gamma as biomarkers in patients with Systemic Lupus Erythematosus and their correlation with disease activity. The Egyptian Rheumatologist.

[21] Vega L, Barbado J, Almansa R, Gonzalez-Gallego R, Rico L, Jimeno A (2010). Prolonged standard treatment for systemic lupus erythematosus fails to normalize the secretion of innate immunity-related chemokines. Eur Cytokine Netw.

[22] Ruchakorn N, Ngamjanyaporn P, Suangtamai T, Kafaksom T, Polpanumas C, Petpisit V (2019). Performance of cytokine models in predicting SLE activity. Arthritis Res Ther..

[23] Mao YM, Zhao CN, Liu LN, Wu Q, Dan YL, Wang DG (2018). Increased circulating interleukin-8 levels in systemic lupus erythematosus patients: a meta-analysis. Biomark Med.

[24] Rodriguez-Rosales YA, Langereis JD, Gorris MAJ, van den Reek J, Fasse E, Netea MG (2021). Immunomodulatory aged neutrophils are augmented in blood and skin of psoriasis patients. J Allergy Clin Immunol.

